# Obesity-Related Pitfalls of Virtual versus True Non-Contrast Imaging—An Intraindividual Comparison in 253 Oncologic Patients

**DOI:** 10.3390/diagnostics13091558

**Published:** 2023-04-26

**Authors:** Henner Huflage, Andreas Steven Kunz, Robin Hendel, Johannes Kraft, Stefan Weick, Gary Razinskas, Stephanie Tina Sauer, Lenhard Pennig, Thorsten Alexander Bley, Jan-Peter Grunz

**Affiliations:** 1 Department of Diagnostic and Interventional Radiology, University Hospital Würzburg, 97080 Würzburg, Germany; 2 Department of Radiation Oncology, University Hospital Würzburg, 97080 Würzburg, Germany; 3 Institute for Diagnostic and Interventional Radiology, Faculty of Medicine, University Hospital Cologne, 50931 Cologne, Germany

**Keywords:** dual-energy CT, dual-source CT, virtual non-contrast, radiation dose, spectral CT, obesity

## Abstract

Objectives: Dual-source dual-energy CT (DECT) facilitates reconstruction of virtual non-contrast images from contrast-enhanced scans within a limited field of view. This study evaluates the replacement of true non-contrast acquisition with virtual non-contrast reconstructions and investigates the limitations of dual-source DECT in obese patients. Materials and Methods: A total of 253 oncologic patients (153 women; age 64.5 ± 16.2 years; BMI 26.6 ± 5.1 kg/m^2^) received both multi-phase single-energy CT (SECT) and DECT in sequential staging examinations with a third-generation dual-source scanner. Patients were allocated to one of three BMI clusters: non-obese: <25 kg/m^2^ (*n* = 110), pre-obese: 25–29.9 kg/m^2^ (*n* = 73), and obese: >30 kg/m^2^ (*n* = 70). Radiation dose and image quality were compared for each scan. DECT examinations were evaluated regarding liver coverage within the dual-energy field of view. Results: While arterial contrast phases in DECT were associated with a higher CTDI_vol_ than in SECT (11.1 vs. 8.1 mGy; *p* < 0.001), replacement of true with virtual non-contrast imaging resulted in a considerably lower overall dose-length product (312.6 vs. 475.3 mGy·cm; *p* < 0.001). The proportion of DLP variance predictable from patient BMI was substantial in DECT (R^2^ = 0.738) and SECT (R^2^ = 0.620); however, DLP of SECT showed a stronger increase in obese patients (*p* < 0.001). Incomplete coverage of the liver within the dual-energy field of view was most common in the obese subgroup (17.1%) compared with non-obese (0%) and pre-obese patients (4.1%). Conclusion: DECT facilitates a 30.8% dose reduction over SECT in abdominal oncologic staging examinations. Employing dual-source scanner architecture, the risk for incomplete liver coverage increases in obese patients.

## 1. Introduction

Dual-energy computed tomography (DECT) has been established in an increasing number of applications for multiple body regions in recent years [1,2,3,4,5,6,7]. However, DECT can result in longer reading times for radiologists due to increased information content [8]. In contrast to single-energy CT (SECT) examinations, DECT allows for material decomposition and semiquantitative analysis based on different X-ray absorption behaviors at two tube voltage settings [9,10]. Frequently used applications in clinical practice include generating virtual non-contrast images, performing iodine quantification and enhancing tissue contrast by composing virtual low-keV monoenergetic images [1,11,12]. True non-contrast examinations facilitate the evaluation of iodine uptake, hence aiding the differentiation of calcified and enhancing lesions [13,14]. Combining pre- and post-contrast scans in the same patient may offer advantages in the determination of lesion vitality, e.g., in regressive malignant lesions, such as metastases or lymph nodes [15]. In addition, the extent of iodine uptake can be color-coded to enhance the visual detection of lesions. Furthermore, using VNC from DECT data instead of true non-contrast studies prevents mismatches between pre- and post-contrast scans due to differences in inspiration depth.

Technical approaches for DECT with energy-integrating detectors include dual-source systems and single-source concepts using fast kilovoltage switching, split-beam techniques or dual-layer detectors [9,16], each bearing specific advantages and disadvantages that must be considered when designing scan protocols. Third-generation dual-source scanners incorporate two tubes positioned 90 degrees apart which are simultaneously operated with different voltages. A typical setting can employ voltages of 100 kVp and 150 kVp with additional beam hardening by a 0.6 mm tin filter [10]. Dual-source designs require an overlap of both beams for spectral applications, significantly limiting the available field of view (FOV) for dual-energy computations. Image data outside of the dual-energy FOV is not covered by the smaller B-tube operating at a higher tube voltage of 150 kVp. In consequence, DECT requires ideal patient positioning within the isocenter, especially if organs of interest are anatomically located in the periphery of the spectral volume.

While the advantages of DECT regarding interpretability and added diagnostic value have been thoroughly proven in numerous studies, direct intraindividual comparisons of clinical DECT and SECT protocols within a large patient cohort using dual-source scanner architecture are still lacking. The aim of this study was to assess the potential for radiation dose reduction in multi-phase contrast-enhanced abdominal DECT and investigate potential implications of patients’ body mass indices on dual-energy coverage.

## 2. Materials and Methods

### 2.1. Study Population

**Figure 1:**
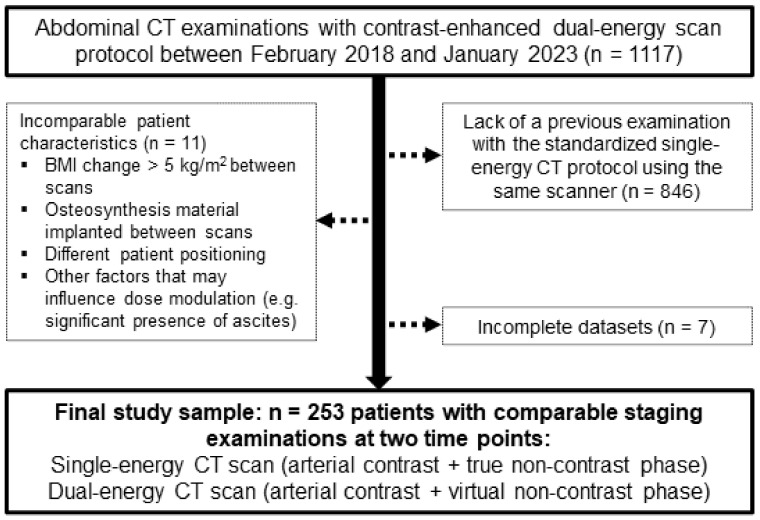
Flow chart for visualization of study inclusions and exclusions.

For inclusion in this retrospective single-center study, 1117 patients who underwent an abdominal DECT scan for oncologic imaging purposes on a third-generation dual-source system (SOMATOM Force; Siemens Healthineers; Forchheim, Germany) between February 2018 and January 2023 were eligible. Lack of a previous SECT study on the same scanner led to the exclusion of 846 patients. Furthermore, 11 patients with incomparable examinations (e.g., BMI change > 5 kg/m^2^ between scans, newly inserted osteosynthetic implants within the scan range, difference in positioning, presence of ascites at one timepoint) and 7 patients with incomplete datasets were excluded from further statistical analyses. The flowchart provided in Figure 1 elucidates inclusion and exclusion criteria, as well as the resulting final population. Permission for this retrospective study was granted by the local institutional review board, which waived the need for additional informed consent (IRB number 20230209 01).

### 2.2. Phantom Scan

For in vitro comparison of scan protocols, a tissue-equivalent CT to electron density calibration phantom (Advanced Electron Density Phantom; Sun Nuclear Corporation; Melbourne, FL, USA) was scanned. The 30 cm × 40 cm phantom possesses an oval shape and an effective diameter of 34.64 cm [17]. Both a SECT and DECT scan protocol were used for image acquisition. Detailed scan parameters of both scan protocols are displayed in Table 1. Standardized regions of interest (ROIs) with a diameter of 20 mm were positioned and image noise was measured in four different tissue inserts (water, fat, lung, and bone). Repeated measurements were averaged across five consecutive slices and image noise was determined by standard deviation of the mean attenuation in Hounsfield units (HU).

### 2.3. Patient Examinations

**Figure 2:**
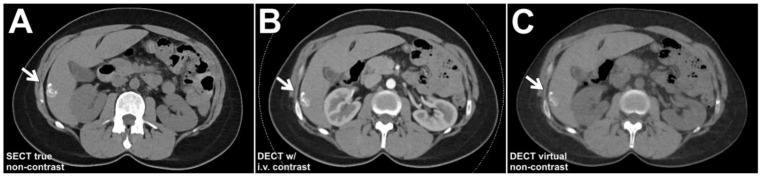
A 42-year-old woman with a history of metastasized breast cancer. A calcified liver lesion (arrows) was visible in the true non-contrast phase of the single-energy baseline CT (SECT; (**A**)). The dual-energy follow-up CT scan with intravenous contrast enhancement (DECT; (**B**)) allows for calculation of virtual non-contrast images that can replace the true non-contrast scan in depicting the sclerotic hemangioma in liver segment 6 (**C**).

Routine SECT patient examinations consisted of a true non-contrast scan of the upper abdomen followed by an arterial contrast phase scan of the same length with a delay of 15 s subsequent to bolus tracking with the ROI placed in the abdominal aorta. In addition, a full-length contrast-enhanced phase of the torso was acquired after another 35 s. The DECT protocol also included a contrast-enhanced arterial phase scan of the upper abdomen with a delay of 15 s and identical parameters for bolus tracking; however, these scans were performed in dual-energy technique. The true non-contrast phase was omitted in DECT in favor of a virtual non-contrast image stack (Figure 2). While acquisition was performed for clinical purposes, respectively, the delayed contrast-enhanced phases of both SECT and DECT examinations were not used for further data analyses in this study. Radiation dose was recorded in the automatically generated patient dose report in the form of CT dose indices by volume (CTDI_vol_) and dose-length products (DLP). The effective radiation dose was estimated by multiplying the DLP with a standardized conversion factor established for imaging of the upper abdomen (k = 0.016 mSv/mGy) [18]. For both scan protocols, reconstructions in the three radiological standard planes were performed using a vendor-specific, soft tissue convolution kernel (Br36; frequency at the 50% and 10% value of the modulation transfer function: ρ_50_ = 3.4/cm, ρ_10_ = 5.4/cm) with strength level 3 of a third-generation iterative reconstruction algorithm (ADMIRE; Siemens Healthineers). For DECT datasets, the standard vendor setting of 0.6 was applied in blended image reconstructions (mixed image data from both tubes); otherwise, the same reconstruction settings were used as previously described for SECT data. After scanner-side image reconstruction, image data were transferred to the clinical PACS system (Merlin; Phönix-PACS; Freiburg, Germany). To obtain a quantitative criterion for image quality, circular ROIs were drawn as large as possible within the intraabdominal fat tissue in the arterial contrast phase image stack of SECT scans (while carefully avoiding other anatomical structures or partial volume effects) and then copied to the arterial phase of contrast-enhanced DECT examinations. Manual adjustments were performed to receive comparable ROI positioning and size in both SECT and DECT scans.

### 2.4. Dual-Energy Coverage of Liver Parenchyma

In order to prepare virtual non-contrast reconstructions of the entire liver from DECT scans, reliable and full coverage of parenchyma within the dual-energy FOV is essential. Using a dual-source CT system, the dual-energy FOV represents the portion of scan volume covered by X-ray beams from both tubes. Measured in the axial plane, the dual-energy FOV of the employed third-generation scanner has a diameter of 35.6 cm; hence, rendering correct positioning for DECT scans is a difficult task. For this study, all DECT scans were evaluated with regard to full coverage of liver parenchyma within the dual-energy FOV. Patients with incomplete coverage were further categorized into three subgroups according to the maximum diameter of tissue outside of the dual-energy FOV on axial slices (<1 cm; 1–2 cm; >2 cm).

### 2.5. Statistical Analysis

Statistical software was used for all data analyses (SPSS Statistics 28, IBM, Armonk, NY, USA). Normal distribution of continuous items was assessed with Q–Q diagrams and Shapiro–Wilk tests. We report demographic variables as means ± standard deviation and radiation dose indicators in the form of median values and interquartile ranges. Metric data was compared using paired student’s *t* tests and one-way analyses of variance with pairwise post hoc tests. In addition, linear regression analyses were conducted to assess the relation between radiation dose, image noise, and patient BMI as an independent variable. Paired data without normal distribution were compared using the Wilcoxon signed rank test. Statistical significance was calculated for each pairwise comparison with Bonferroni correction of *p* values. A type I error < 0.05 was deemed indicative of statistical significance.

## 3. Results

### 3.1. Patient Sample

Adhering to the predefined inclusion and exclusion criteria, the study population consisted of 253 patients (153 women) with a mean age of 64.5 ± 16.2 years. Examinations were performed for various oncological diseases (43.5% melanoma, 24.1% breast carcinoma, 8.7% pancreatic carcinoma, 7.5% renal carcinoma, 4.7% liver carcinoma, 3.9% adrenal carcinoma, 7.5% miscellaneous). Mean patient weight and height at baseline were 77.1 ± 16.8 kg and 170 ± 9 cm, respectively, corresponding to a mean BMI of 26.6 ± 5.1 kg/m^2^. For further analyses, patients were allocated to one of three groups based on their BMI at baseline imaging: no obesity, <25 kg/m^2^ (*n* = 110); pre-obesity, 25–29.9 kg/m^2^ (*n* = 73); obesity, >30 kg/m^2^ (*n* = 70). Detailed demographic data for each BMI cluster are displayed in Table 2. The median interval between CT examinations was 375 days (interquartile range 200–581 days).

### 3.2. Radiation Dose

#### 3.2.1. Phantom Scan

Prior to application in human patients, both scan protocols were performed on the aforementioned dosimetry phantom. For SECT scans, the automatic tube voltage selection and tube current modulation were activated, resulting in a CTDI_vol_ of 10.0 mGy at 100 kVp. In contrast, the DECT scan protocol using 100/Sn 150 kVp lead to a CTDI_vol_ of 15.5 mGy. Median image noise in water (18.8 HU versus 13.3 HU), fat (14.8 HU versus 11.8 HU), lung (26.3 HU versus 23.2 HU), and bone tissue (19.1 HU versus 14.7 HU) was substantially higher in SECT versus DECT examinations (all *p* ≤ 0.043).

#### 3.2.2. Patient Examinations

**Figure 3:**
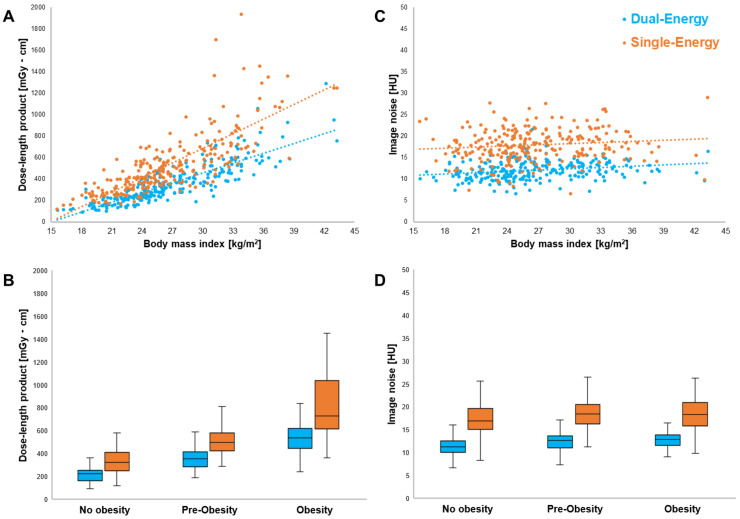
Linear regression analysis and scatter plots revealed the influence of patient BMI on the DLP of DECT (blue) and SECT scans (orange) (**A**). Note the increasing radiation dose difference between SECT and DECT in obese patients (**B**). In contrast, the level of image noise in abdominal fat tissue was largely independent of BMI for both DECT (R^2^ = 0.057) and SECT (R^2^ = 0.015) (**C**). Noise in SECT was constantly higher in all three BMI clusters (**D**).

While CTDI_vol_ of arterial contrast phases was higher in DECT compared to SECT scans (11.1 mGy versus 8.1 mGy; *p* < 0.001), combined DLP of arterial and unenhanced imaging was substantially lower in DECT examinations (312.6 mGy·cm versus 475.3 mGy·cm; *p* < 0.001) based on the replacement of a true non-contrast with a virtual non-contrast phase. Accordingly, the effective radiation dose associated with the DECT scan protocol was 30.8% lower than that of SECT studies (2.2 mSv, interquartile range 1.3–4.0 mSv; *p* < 0.001). The proportion of DLP variance predictable from patient BMI was substantial in both DECT (R^2^ = 0.738) and SECT (R^2^ = 0.620). Compared with DECT, the DLP of SECT examinations showed a stronger increase in obese patients with high BMI (*p* < 0.001). In contrast, the influence of patient BMI on the level of image noise was small for both DECT (R^2^ = 0.057) and SECT (R^2^ = 0.015) (Figure 3). While image noise in abdominal fat tissue was generally higher in SECT examinations (17.8 HU versus 11.9 HU; *p* < 0.001), the difference between DECT and SECT remained constant among the three BMI clusters (*p* = 0.593). Detailed subgroup analyses of radiation dose and image noise measurements are presented in Table 3.

### 3.3. Dual-Energy Coverage of Liver Parenchyma

**Figure 4:**
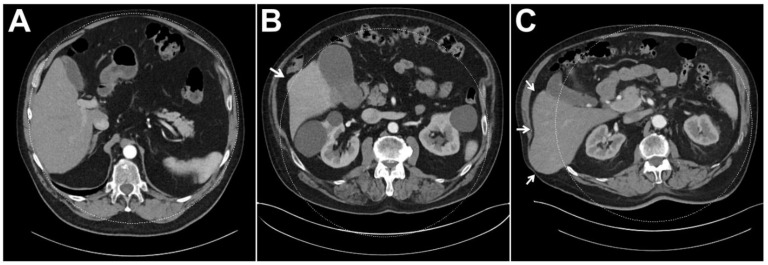
In dual-source CT, reliable coverage of liver parenchyma within the dual-energy FOV is essential for diagnostic assessment as virtual non-contrast information is not available for organ portions that are not exposed to both X-ray spectra. While the liver was included within the dual-energy FOV in its entirety in the vast majority of patients (**A**), results indicate that marginal lack of coverage (**B**) and considerable misalignment (**C**) are more common in obese patients. (The arrow indicated liver parenchyma not covered by the DE-FOV).

Of the 253 DECT scans analyzed in this study, complete liver parenchyma coverage within the dual-energy FOV was achieved in 238 studies (94.1%). Table 4 summarizes the frequency of incomplete coverage in the three BMI clusters. Notably, incomplete coverage was most common in the obese subgroup (17.1%) compared with the non-obese (0%) and pre-obese cluster (4.1%). Figure 4 displays exemplary cases of incomplete liver parenchyma coverage.

## 4. Discussion

With this study, we demonstrate the dose reduction potential of an abdominal dual-source dual-energy CT protocol over standard multiphase single-energy CT in various oncologic diseases. For staging examinations requiring an arterial contrast phase (e.g., melanoma, breast carcinoma, pancreatic carcinoma, renal carcinoma, liver carcinoma, and adrenal carcinoma among others) the replacement of a true with a virtual non-contrast phase facilitated dose saving of 30.8% over all intraindividual patient comparisons. In cluster-based analysis, radiation dose reduction over conventional single-energy CT was particularly high in obese individuals. It must be noted, however, that the risk of incomplete organ coverage and therefore insufficient diagnostic yield increases considerably in this patient group.

In order to guarantee sufficient image quality independent of patient weight, the fixed combination of tube voltages 100 kVp/Sn 150 kVp was chosen for DECT examinations in this study. While individual studies suggest that a weight-dependent change in the voltage of the A-tube may improve the spectral separation and lower the image noise, irrespective of patient size [10,19], it should be mentioned that the automated selection of tube potential is not applicable for dual-energy scan protocols (e.g., 80 kVp/Sn 150 kVp vs. 100 kVp/Sn 150 kVp), although each tube current is independently modulated by the automatic exposure control [20]. Since a higher CTDI_vol_ was observed for DECT in CT-to-electron density calibration phantom measurements and based on the fact that the A-tube is operated at the maximum of 100 kVp, the helical pitch factor was reduced to 0.6 to ensure diagnostic quality in all examinations. Of note is that the ratio between both tube currents depends substantially on the patient diameter, hence making it difficult to reliably predict the noise level in dependence of the radiation burden [20]. Further investigating this aspect, previous studies have reported a smaller increase in size-specific dose estimates in relation to the increase in effective patient diameter in DECT compared to conventional SECT [20]. This observation suggests that weight-adapted scan protocols could be beneficial in larger patient diameters [19]. Since an increase in image noise was previously postulated for virtual non-contrast images [21], we decided to employ a higher individual CTDI_vol_ for the contrast-enhanced dual-energy scan in this study. Consistent with the literature, significant dose savings were achieved nonetheless, compared with SECT containing a true non-contrast phase [22,23]. Purysko et al. reported a linear correlation between radiation dose and patient size with SECT and a steeper dose increase in obese patients than with DECT. Despite differences in B-tube potential (140 kVp versus 150 kVp) and tin filter thickness (0.4 mm versus 0.6 mm) between second and third dual-source scanner generations, the results are in line with the findings in our study [10,24].

In clinical routine, the achievable dose savings heavily depend on the patient cohort and the individual protocol settings. Particularly in comparison with low kV levels, a direct comparison of image quality may show a disadvantage of DECT in terms of image contrast [25]. Additionally, DECT protocols require a change in the radiologist’s workflow, as most advantages of dual-energy-based imaging only become evident when reading the associated virtual monoenergetic and/or virtual non-contrast datasets in addition [25]. The extent to which DECT protocols are implemented for clinical staging examinations should be weighed up by the respective radiologist. Off-center positioning of obese patients also has potential downsides such as reduced image quality and suboptimal dose efficiency. Therefore, it cannot be recommended in general. For patients in which previous examinations are available, screening of pre-existing datasets with regard to habitus and other factors, e.g., abdominal wall hernias, may be a viable method to avoid incomplete coverage of liver parenchyma within the dual-energy FOV. In essence, individual protocol setups must be carefully balanced and tested before dispensing conventional SECT in favor of DECT examinations. This study proves that obese patients do benefit from DECT protocols in terms of reduced radiation dose exposure, albeit at the risk of insufficient diagnostic yield due to incorrect patient positioning with partial exclusion of organ parenchyma from the dual-energy FOV. Further studies to account for the altered biological effectiveness at different kVp between SECT and dose equivalent DECT examinations would be desirable. Regardless of BMI cluster, our results concur with those of De Cecco et al., who reported a relative dose saving of 32.9% by omitting the true non-contrast scan in a multiphase DECT examinations of the liver.

Several limitations must be mentioned regarding the study’s methodology: First, repeated examinations of the same patient at the same timepoint could not be realized due to ethical and radiation protection concerns. Therefore, sequential staging studies were used for intraindividual comparisons instead. Second, slight variations in patient positioning and scan lengths may have affected the resulting dose values. Third, additional dose reduction may have been achievable by lowering the reference tube current for the DECT protocol to the level of SECT scans. However, due to higher inherent image noise in virtual non-contrast phases, we deemed the choice of tube current reasonable. Since proper comparisons of image contrast would have required identical amount and flow of contrast medium in each scan as well as comparable blood circulation times, such quantifications were omitted in favor of noise measurements [20]. Finally, the equipment of one vendor was employed for patient examinations, hence, potentially limiting the generalizability of the reported results.

## 5. Conclusions

By replacing the true with a virtual non-contrast phase, abdominal dual-energy CT allows for substantial dose reduction over single-energy CT in oncologic staging examinations. Employing dual-source scanner architecture, the risk for incomplete coverage of liver parenchyma increases considerably in obese patients.

## Figures and Tables

**Table 1:** Scan parameters.

	Single-Energy CT	Dual-Energy CT
Scanner	SOMATOM Force; Siemens Healthineers	SOMATOM Force; Siemens Healthineers
Phase	Arterial phase (+true non-contrast phase)	Arterial phase (+virtual non-contrast phase)
Automated dose modulation	CARE Dose 4D, CARE kV	CARE Dose 4D
Tube voltage [kVp]	80–150	100/Sn 150
Reference tube current [mAs]	120	200/100
Pitch	0.9	0.6
Rotation time [s]	0.5	0.5
Collimation [mm]	192 × 0.6 mm	128 × 0.6 mm
Iterative reconstruction	ADMIRE, strength level 3	ADMIRE, strength level 3
Convolution kernel [1/cm]	Br36 (ρ_50_ = 3.4/cm; ρ_10_ = 5.4)	Br36 (ρ_50_ = 3.4/cm; ρ_10_ = 5.4)
Contrast agent	Weight-adapted; iodine amount 350 mg/mL	Weight-adapted; iodine amount 350 mg/mL

Note: ADMIRE: advanced modelled iterative reconstruction; ρ_50_/ρ_10_ = frequency at the 50%/10% value of the modulation transfer function.

**Table 2:** Patient population.

	All Patients	No Obesity	Pre-Obesity	Obesity
	*n* = 253	*n* = 110	*n* = 73	*n* = 70
		BMI < 25.0 kg/m^2^	BMI 25.0–30.0 kg/m^2^	BMI > 30.0 kg/m^2^
Patient characteristics				
Sex [women/men]	153/100 (60.5%/39.5%)	80/30 (72.7%/27.3%)	28/45 (38.4%/61.6%)	45/25 (64.3%/35.7%)
Age [years]	64.5 ± 16.2	63.8 ± 17.7	66.6 ± 12.2	63.6 ± 17.3
Weight [kg]	77.1 ± 16.8	62.8 ± 9.5	81.2 ± 9.1	95.2 ± 11.3
Height [cm]	170 ± 9	168 ± 9	173 ± 9	169 ± 8
Body mass index [kg/m^2^]	26.6 ± 5.1	22.2 ± 2.2	27.0 ± 1.3	33.4 ± 2.9
Oncologic disease				
Melanoma	110 (43.5%)	32 (29.1%)	38 (52.1%)	40 (57.1%)
Breast carcinoma	61 (24.1%)	32 (29.1%)	14 (19.2%)	15 (21.4%)
Pancreatic carcinoma	22 (8.7%)	13 (11.8%)	4 (5.5%)	5 (7.1%)
Renal carcinoma	19 (7.5%)	9 (8.2%)	8 (11.0%)	2 (2.9%)
Liver carcinoma	12 (4.7%)	6 (5.5%)	5 (6.8%)	1 (1.4%)
Adrenal carcinoma	10 (3.9%)	4 (1.6%)	1 (1.4%)	5 (7.1%)
Miscellaneous	19 (7.5%)	14 (12.7%)	3 (4.1%)	2 (2.9%)

Note: Demographic variables are reported as mean ± standard deviation, whereas cancer history is displayed as absolute frequencies (percentages).

**Table 3:** Image noise and radiation dose comparisons.

	All Patients	No Obesity	Pre-Obesity	Obesity
Single-energy CT				
Image noise in abdominal fat tissue [HU]	17.8 (15.7–20.1)	16.9 (15.2–19.6)	18.4 (16.6–20.5)	18.4 (15.9–20.9)
CTDI_vol_ of true non-contrast phase [mGy]	8.3 (6.2–11.0)	6.0 (5.0–7.2)	8.7 (7.5–10.1)	12.8 (10.8–16.7)
CTDI_vol_ of arterial phase [mGy]	8.1 (6.1–10.9)	6.0 (4.8–7.2)	8.6 (7.3–10.2)	12.9 (10.6–16.3)
DLP of true non-contrast phase [mGy·cm]	240.1 (180.3–333.1)	170.8 (135.5–214.1)	258.7 (224.7–301.2)	380.3 (319.4–514.3)
DLP of arterial phase [mGy·cm]	228.7 (161.1–307.8)	156.8 (120.6–194.2)	247.0 (190.8–281.8)	354.9 (293.6–482.0)
DLP of true non-contrast + arterial phase [mGy·cm]	475.3 (343.4–648.9)	325.6 (252.6–409.5)	498.2 (426.2–575.6)	729.9 (619.6–1026.2)
Dual-energy CT				
Image noise in abdominal fat tissue [HU]	11.9 (10.6–13.6)	11.2 (10.1–12.5)	12.7 (11.0–13.6)	12.9 (11.6–13.9)
CTDI_vol_ of arterial phase [mGy]	11.1 (8.5–15.5)	8.4 (6.9–9.5)	12.4 (10.7–14.5)	17.8 (16.0–20.8)
DLP of arterial phase [mGy·cm]	312.6 (231.5–450.6)	224.6 (165.4–253.9)	356.5 (287.1–414.8)	536.5 (450.3–610.7)
Single-energy CT versus dual-energy CT				
Absolute noise difference [HU]	5.7 (4.0–7.6)	5.8 (3.9–7.8)	5.7 (4.1–7.3)	5.9 (4.1–7.2)
DLP difference [mGy·cm]	134.6 (78.9–249.9)	104.0.6 (68.3–165.0)	134.5 (85.4–215.2)	248.2 (121.5–377.6)

Note: Indicators of radiation dose are reported as median values (interquartile ranges). CTDI_vol_—volume CT dose index; DLP—dose-length product.

**Table 4:** Liver parenchyma coverage in the field of view of dual-energy CT examinations.

	All Patients	No Obesity	Pre-Obesity	Obesity
Full coverage of liver parenchyma in the DE-FOV	238 (94.1%)	110 (100%)	70 (95.9%)	58 (82.9%)
<1 cm of liver parenchyma outside of the DE-FOV	5 (2.0%)	0 (0%)	0 (0%)	5 (7.1%)
1–2 cm of liver parenchyma outside of the DE-FOV	5 (2.0%)	0 (0%)	3 (4.1%)	2 (2.9%)
>2 cm of liver parenchyma outside of the DE-FOV	5 (2.0%)	0 (0%)	0 (0%)	5 (7.1%)

Note: Items are reported as absolute frequencies (percentages). DE-FOV—dual-energy field of view.

## Data Availability

Data are made available upon reasonable request.

## Abbreviations

CTDI_vol_	Volume computed tomography dose index
DECT	Dual-energy computed tomography
FOV	Field of view
HU	Hounsfield units
ROI	Region of interest
SECT	Single-energy CT
